# Towards clinical adherence monitoring of oral endocrine breast cancer therapies by LC-HRMS—method development, validation, comparison of four sample matrices, and proof of concept

**DOI:** 10.1007/s00216-024-05244-6

**Published:** 2024-03-15

**Authors:** Cathy M. Jacobs, Julia C. Radosa, Lea Wagmann, Julia S. M. Zimmermann, Askin C. Kaya, Aylin Aygün, Tatjana Edel, Lisa Stotz, Mohamed Ismaeil, Erich-Franz Solomayer, Markus R. Meyer

**Affiliations:** 1 Department of Experimental and Clinical Toxicology, Institute of Experimental and Clinical Pharmacology and Toxicology, Center for Molecular Signaling (PZMS), Saarland University, Homburg, Germany; 2 Department of Gynecology, Obstetrics and Reproductive Medicine, Saarland University Hospital, Homburg, Saarland Germany

**Keywords:** Adherence monitoring, Sample matrices, Breast cancer, LC-HRMS, Bioanalysis

## Abstract

Oral endocrine therapies (OET) for breast cancer treatment need to be taken over a long period of time and are associated with considerable side effects. Therefore, adherence to OET is an important issue and of high clinical significance for breast cancer patients’ caregivers. We hypothesized that a new bioanalytical strategy based on liquid chromatography and high-resolution mass spectrometry might be suitable for unbiased adherence monitoring (AM) of OET. Four different biomatrices (plasma, urine, finger prick blood by volumetric absorptive microsampling (VAMS), oral fluid (OF)) were evaluated regarding their suitability for AM of the OET abemaciclib, anastrozole, exemestane, letrozole, palbociclib, ribociclib, tamoxifen, and endoxifen. An analytical method was developed and validated according to international recommendations. The analytical procedures were successfully validated in all sample matrices for most analytes, even meeting requirements for therapeutic drug monitoring. Chromatographic separation of analytes was achieved in less than 10 min and limits of quantification ranged from 1 to 1000 ng/mL. The analysis of 25 matching patient samples showed that AM of OET is possible using all four matrices with the exception of, e.g., letrozole and exemestane in OF. We were able to show that unbiased bioanalytical AM of OET was possible using different biomatrices with distinct restrictions. Sample collection of VAMS was difficult in most cases due to circulatory restraints and peripheral neuropathy in fingers and OF sampling was hampered by dry mouth syndrome in some cases. Although parent compounds could be detected in most of the urine samples, metabolites should be included when analyzing urine or OF. Plasma is currently the most suitable matrix due to available reference concentrations.

**Graphical Abstract:**

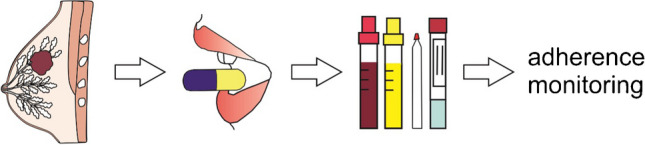

**Supplementary Information:**

The online version contains supplementary material available at 10.1007/s00216-024-05244-6.

## Introduction

Around 2.3 million women are diagnosed with breast cancer annually [1]. Adjuvant oral endocrine therapy (OET) is recommended for women with hormone receptor-positive breast cancer and studies showed an improved disease-free and overall survival for patients treated with OET compared to patients who do not take OET. Clinical guidelines recommend at least a treatment period of 5 years [2]. Nonadherence, a failure to take medication as prescribed by the physician or the discontinuation of medication prior to the prescribed duration, is a clinical challenge for caregivers treating breast cancer patients after primary therapy, in breast cancer treatment [2]. In chronic diseases, evidence indicates that only about half of patients are adherent towards their medication [3]. In general, patients rated poorer in health were found to be more adherent to treatment [4]. Therefore, nonadherence is expected to be less of an issue in cancer patients since patients are considered as highly motivated due to the seriousness of their disease. However, in breast cancer patients receiving OET, concerns of nonadherence have been identified [3]. Studies showed that despite the demonstrated benefits of OET, only about half of breast cancer patients are adherent to their prescribed regimen and two-thirds discontinue therapy before the recommended 5 years [2, 5]. Factors associated with poor adherence include patients’ perception of an unfavorable risk/benefit ratio of the therapy, adverse events endured, depressive symptoms, and medication costs [3]. The accurate assessment of adherence to OET is necessary for an effective and efficient treatment of patients [6, 7]. Hereby, the direct adherence assessment via bioanalytical measurements, e.g., liquid chromatography–mass spectrometry (LC–MS) of OET in different sample matrixes, can be used [7]. LC–MS-based analysis is sensitive and selective [7, 8], and it has been shown for other chronic diseases that LC–MS-based adherence monitoring can improve patients’ level of adherence [9]. Several sample matrices are available for LC–MS-based adherence monitoring. Venous blood and urine may be most used, however, alternative matrices, e.g., volumetric absorptive microsampling (VAMS) and oral fluid (OF), were also successfully applied for adherence monitoring [10–12]. Each sample matrix has its own advantages and disadvantages for adherence assessment which can be found elsewhere [7]. Briefly, reference concentrations are mostly available for blood plasma; however, the sampling procedure is invasive and must be performed by a healthcare professional [13]. Urine is easily accessible and often described as the gold standard to detect nonadherence due to a longer time window available for drug detection compared to blood [7, 14–16]; however, urinary concentrations are affected by, e.g., urinary flow, frequency of bladder emptying, and the drug’s pharmacokinetic profile. On one hand, the mostly qualitative nature of analysis using urine may lead to overestimation of adherence due to the excretion of drugs exceeding the dosing interval manyfold [7, 17]; on the other hand, some drugs are mainly excreted in feces, e.g., abemaciclib (81%), palbociclib (74%), and ribociclib (69%) which can lead to an underestimation of adherence [18]. VAMS where a small drop of finger prick blood (FPB) is collected is minimally invasive and trained patients can perform the sampling procedure themselves at home and send the sample via mail to the laboratory. However, due to the small sample volume, sensitive analysis techniques are required and reference concentrations for plasma cannot always be transferred to capillary whole blood. OF sampling is noninvasive and suitable for at-home sampling; however, drug concentrations can be influenced by amongst others salivary flow and contaminations in the oral cavity. Furthermore, there is a lack of available reference concentrations for OF.

This study aimed to develop and evaluate strategies using four different sample matrices, plasma, urine, VAMS, and OF by means of LC-high-resolution (HR) MS for adherence monitoring of eight commonly prescribed OETs. The workflows should be validated according to international guidelines [19, 20]. Furthermore, a proof of concept was performed to demonstrate the clinical applicability and to reveal possible limitation of the different sample matrices. Methods for the quantification of some OET in plasma or dried blood spots are available in the literature [21–24]. However, to the best of our knowledge, this is the first method covering the cyclin-dependent kinase inhibitors abemaciclib, palbociclib, and ribociclib, the estrogen receptor modulators endoxifen and tamoxifen, and the aromatase inhibitors anastrozole, letrozole, and exemestane in four different sample matrices.

## Materials and methods

### Chemicals and materials

The internal standards (IS) abemaciclib-d_8_, anastrozole-^13^C_4_, letrozole-^13^C_2_, palbociclib-d_8_, ribociclib-d_6_, and tamoxifen-d_5_ were purchased from Alsachim (Illkirch Graffenstaden, France); exemestane-^13^C,d_3_ from LGC (Luckenwalde, Germany); the compound endoxifen (E/Z, 1:1)-d^5^ was purchased from Toronto Research Chemicals (Toronto, Canada) and anastrozole, exemestane, letrozole, and palbociclib from LGC (Luckenwalde, Germany); abemaciclib, ribociclib, and tamoxifen from Toronto Research Chemicals (Toronto, Canada); (E/Z,1:1)-endoxifen and (Z)-endoxifen from Sigma-Aldrich (St. Louis, USA). All other chemicals (LC–MS grade or analytical grade) were from VWR (Darmstadt, Germany). Mitra VAMS with a 10 µL absorbing tip were purchased from Neoteryx (Torrance, USA). Quantisal devices were purchased from Abbott Rapid Diagnostics (Köln, Germany). Blank blood stabilized with ethylenediaminetetraacetic acid (EDTA) and blank urine used for development and validation of the procedure was submitted to the authors’ laboratory for regular toxicological analysis and handled in accordance with the institutional protocol and regulations concerning data privacy and sample handling. Blank OF was provided by drug-free volunteers. FPB-loaded VAMS, matching EDTA-stabilized blood samples, urine samples, OF samples collected with the Quantisal device, and medication plans for the proof-of-concept study were collected from volunteering patients with breast cancer between February 2022 and Mai 2023 as part of their regular consultation at Saarland University Hospital, Homburg, Germany. All volunteers provided written informed consent and the local ethics committees approved the study (No. 171/21).

### Calibrators, quality controls, internal standards

Stock solutions of each compound were prepared at a concentration of 1 mg mL^−1^ in methanol except for palbociclib and palbociclib-d_8_ at a concentration of 0.5 mg mL^−1^ in DMSO. The IS solution contained 500 ng mL^−1^ ribociclib-d_6_, 200 ng mL^−1^ abemaciclib-d_8_, letrozole-^13^C_2_, 50 ng mL^−1^ anastrolzole-^13^C_4_, palbociclib-d_8_, tamoxifen-d_5_, 10 mg mL^−1^ endoxifen-d_5_, exemestane-^13^C,d_3_ in methanol. All solutions were stored in amber glass vials at − 20 °C. The quality control (QC) working solutions were prepared by spiking the stock solutions in methanol and the final concentrations in the matrix are shown in Table 1. To preserve the nature of the matrix, the volume of the spiked solution did not exceed 5% of the total matrix volume [25]. To prepare QC samples, 10 µL working solution was added to 190 µL plasma, urine, or blank EDTA-blood, respectively; 100 µL QC working solution was added to 1900 µL blank OF. Human whole EDTA-stabilized blood and plasma were incubated for 30 min at 37 °C and 1500 rpm using a Thermoshaker Pro (CellMedia, Elsteraue, Germany) to allow plasma-protein binding and/or diffusion into red blood cells [11]. VAMS tips were loaded by holding them onto the surface of whole blood until completely soaked with an additional waiting time of 2 s [26]. VAMS tips were dried at room temperature (24 °C) for at least 3 h before sample preparation [27]. A volume of 1000 µL spiked blank OF was given onto the Quantisal swab; the swab was placed into the buffer solution and shaken for 3 h at 1500 rpm, at room temperature (24 °C) using a ThermoMixer C (Eppendorf, Hamburg, Germany).

**Table 1:** Listing of steady-state concentrations (ng mL^−1^), minimal (min) or trough concentrations (ng mL^−1^), and maximal (max) concentrations (ng mL^−1^) of oral endocrine therapies in plasma; final concentrations (ng mL^−1^) of analytes in matrix used for quality controls (QC); corresponding isotope-labeled internal standard (IS); ranges covered in the different sample matrices (ng mL^−1^). (*n.a.* not available, *VAMS* volumetric absorptive microsampling, *OF* oral fluid)

			ng mL^−1^									Range covered, ng mL^−1^			
Analyte	Half life, h	Dose, mg	Steady-state	*C*_trough/min_	*C*_max_	Reference	QC1	QC2	QC3	QC4	IS	Plasma	VAMS	OF	urine
Abemaciclib	17–38	150	n.a	176	249	[18]	50	150	250	450	Abemaciclib-d_8_	50–500	50–500	50–500	50–500
Anastrozole	40–50	1	59	33	n.a	[28, 29]	10	30	50	90	Anastrozole-^13^C_4_	10–100	10–100	10–100	10–100
Endoxifen	49–68	20	146	n.a	n.a	[30]	1	3	50	90	Endoxifen-d_5_	1–100	50–100 (#3–100)	3–100 (#1–100)	1–100
Endoxifen as tamoxifen metabolite	n.a	n.a	11–16	n.a	n.a	[30]	1	3	50	90	Endoxifen-d_5_	1–100	50–100 (#3–100)	3–100 (#1–100)	1–100
Exemestane	10–30	25	n.a	4.1	19	[28, 29]	10	30	50	90	Exemestane-^13^C, d_3_	(#10–100)	10–100	30–100	(#10–100)
Letrozole	24–130	2.5	n.a	88	133	[28, 29]	50	150	250	450	Letrozole-^13^C_2_,^15^N_2_	50–500	150–500 (#50–500)	n.a	150–500 (#50–500)
Palbociclib	24–34	125	n.a	47	97	[18]	10	30	50	90	Palbociclib-d_8_	10–100	10–100	30–100 (#10–100)	10–100
Ribociclib	30–55	600	n.a	457	1680	[18]	100	300	500	950	Ribociclib-d_6_	100–1000	100–1000	100–1000	100–1000
Tamoxifen	96–168	20	122	10	n.a	[28–30]	10	30	50	90	Tamoxifen-d_5_	10–100	30–100 (#10–100)	10–100	10–100

^#^ range for qualitative adherence assessment

### Sample preparation—plasma and urine

A volume of 200 µL plasma or urine was transferred into a 1.5 mL reaction tube and 20 µL IS solution followed by 1000 µL acetonitrile (ACN) was added. Samples were shaken for 5 min at room temperature (24 °C) and 1500 rpm using a Thermoshaker Pro and centrifuged at 15,000 × *g* for 5 min before a volume of 1000 µL supernatant was transferred into a LC vial and dried completely at 70 °C under a gentle stream of nitrogen. Afterwards, samples were reconstituted in 50 µL of mobile phases A and B (1:1, *v/v*) and a volume of 5 µL was injected into the LC-HRMS system to be analyzed as described in “Instruments and settings.”

### Sample preparation—volumetric absorptive microsampling tip

The whole blood-soaked and dried VAMS tip was transferred into a 1.5 mL reaction tube and 10 µL IS solution and 500 µL ACN were added. Samples were shaken for 30 min at 37 °C and 1500 rpm using a Thermoshaker Pro and centrifuged at 15,000 × *g* for 5 min before a volume of 470 µL supernatant was transferred into an LC vial and dried completely at 70 °C under a gentle stream of nitrogen. Afterwards, samples were reconstituted in 50 µL of mobile phases A and B (1:1, *v/v*) and a volume of 5 µL was injected into the LC-HRMS system to be analyzed as described in “Instruments and settings.”

### Sample preparation—oral fluid

A volume of 100 µL Quantisal-buffer solution was transferred into a 1.5 mL reaction tube and 10 µL IS solution and 50 µL ACN were added. Samples were shaken for 5 min at room temperature (24 °C) and 1500 rpm using a Thermoshaker Pro and centrifuged at 15,000 × *g* for 5 min. The supernatant was transferred into a LC vial and a volume of 5 µL was injected into the LC-HRMS system to be analyzed as described in “Instruments and settings.”

### Method development

In preliminary studies, chromatographic separation of analytes was tested using different columns. This included a ThermoFisher Scientific (TF, Dreieich, Germany) Accucore Phenyl-Hexyl column (100 mm × 2.1 mm, 2.6 µm particle size), a TF Hypersil GOLD C18 (100 mm × 2.1 mm, 1.9 µm particle size), a Waters (MA, USA) AQUITY UPLC BEH C18 column (100 × 2.1 mm, 1.7 µm), a Macherey–Nagel (Düren, Germany) HILIC Nucleodur column (125 mm × 3 mm, 3 µm), and a Merck (Darmstadt, Germany) SeQuant ZIC HILIC column (150 mm × 2.1 mm, 3.5 µm). Also, different combinations of mobile phases were tested for best peak separation and peak shape as well as different scan modes for best analyte detection and peak description. Hereby, full MS, full MS with data-dependent MS^2^ using an inclusion list with masses of interest, targeted single ion monitoring (SIM), and parallel reaction monitoring (PRM) mode were compared. A total of five sample preparations were compared using four different concentration levels of OET for each sample matrix. Sample preparation included precipitation with different mixtures of methanol:ACN and liquid–liquid extractions, amongst others with chlorbutanol. Extractions were evaluated for the highest and reproducible peak areas.

### Instruments and settings

All samples were analyzed using a TF Dionex UltiMate 3000 Rapid Separation LC system consisting of a degasser, a quaternary pump, and a UltiMate autosampler, coupled to a TF Q-Exactive Plus mass spectrometer system equipped with a heated electrospray ionization (HESI)-II source. Mass calibration was done prior to analysis according to the manufacturer’s recommendations using external mass calibration (Pierce ESI Negative Ion Calibration Solution, TF). Gradient elution was performed on a TF Accucore Phenyl-Hexyl column (100 mm × 2.1 mm, 2.6 µm particle size). Mobile phase A consisted of 2 mM aqueous ammonium formate containing formic acid (0.1%, *v/v*, pH 3), and mobile phase B consisted of 2 mM aqueous ammonium formate with ACN:methanol (50:50, *v/v*) plus formic acid (0.1%, *v/v*), and water (1%, *v/v*). The gradient was set as follows: 0–0.8 min hold 99% A, 0.8–1 min from 99% A to 70% A, 1–3 min hold 70% A, 3–7.5 min 70% A to 1% A, 7.5–8.5 min hold 1% A, and 8.5–9 hold 99% A. The flow rate was set as follows: 0–7.5 min 0.5 mL min^−1^, 7.5–8.5 min from 0.5 to 0.8 mL min^−1^, 8.5–9 min from 0.8 to 0.5 mL min^−1^. Chromatography was performed at 40 °C. The HESI-II source conditions were as follows: ionization mode, positive; sheath gas flow rate, 60 arbitrary units (AU); auxiliary gas flow rate, 10 AU; spray voltage 4.00 kV; auxiliary gas heater temperature, 320 °C; ion transfer capillary temperature, 320 °C; and S-lens RF level 60.0.

Mass spectrometry analysis was performed using full MS in positive mode. The settings for full MS data acquisition were as follows: resolution, 35,000; automatic gain control (AGC) target 1e^6^; maximum injection time (IT) 100 ms; scan range, 250 to 650 m*/z*; high-energy collisional dissociation (HCD) with normalized collision energy (NCE), 35 e.V. TF Xcalibur Qual Browser software version 2.2 was used for data evaluation. Ion masses (*m/z*) used for peak detection are represented in the electronic supplementary material (ESM) in Table S1.

### Method validation

The method was validated according to the ICH guideline M10 on bioanalytical method validation and study sample analysis or the Guideline on bioanalytical Method Validation of the European Medicines Agency (EMA) [19, 20]. TF Xcalibur Quan browser version 2.2 and Microsoft (Redmond, USA) Excel version 16 were used to perform the statistical evaluation.

Desired quantification ranges were chosen according to available minimal concentrations or trough concentrations, maximal concentrations, and steady-state concentrations of OET (see Table 1). QC level 1 represents the desired lower limit of quantification (LLOQ), QC level 2 was set within three times the desired LLOQ, QC level 3 represents the middle of the desired quantification range, and QC level 4 is at least at 75% of the desired upper limit of quantification (ULOQ).

To test for selectivity of the method, blank plasma, blank urine, blank FPB-soaked VAMS, and blank OF samples, each from six drug-free individuals was analyzed. Processed samples were analyzed for peak interferences with analytes and IS or possible false positive results. Carry-over was tested for each matrix by injecting two extracted blank matrix samples after analysis of the QC level 4 (see Table 1) (*n* = 3). Interfering signals of analytes in the blank matrix for selectivity and carry-over testing should be < 20% of the LLOQ and < 5% of the IS [20].

Quantification was performed via the relative response factor (RRF) of the corresponding isotope-labeled IS, requiring no calibration curves. The unknown concentration in patient samples can be directly calculated using internal calibration via analyte/IS peak area ratio and a predetermined matrix-dependent response factor [31] (ESM Equation S1, Table S1). For endoxifen and its corresponding IS, the sum of the peak areas of E and Z isomers was used for the quantification. The correction factor for the quantification via RRF was determined in each sample matrix at QC levels 1–4 (*n* = 6) (see Table 1) by Equation S2 in ESM. Afterwards, the mean value of the determined RRF for each matrix was used for quantification. Concentrations of QC levels 1–4 in the matrix are represented in Table 1. QCs were prepared by spiking a blank matrix with four individual QC solutions. The sample preparation was performed as described above.

Four QC levels and their back-calculated QC concentrations compared to nominal values were used to assess accuracy and precision. Five repeat samples of each QC level (obtained within a single run) were used to determine the within-run accuracy and precision and five sample replicates of each QC level on three runs (analyzed on three different days) to determine between-run data. Values were considered sufficient when mean concentrations of QCs were within ± 15% of nominal values (± 20% for LLOQ) and CVs within 15% (20% for LLOQ). CV for within-run precision was calculated by dividing the standard deviation of the daily mean (3 days, *n* = 5) by the total mean.

The matrix factor (MF), the IS normalized matrix factor (IS-MF), and the IS normalized recovery (IS-RE) were investigated using blank matrix samples of individual donors at QC level 2 and QC level 4 (Table 1). In the case of VAMS tips as a sample matrix, EDTA-stabilized whole blood with a hematocrit of 40% (*n* = 6) was used to load the VAMS tips. The MF, IS-MF, and IS-RE were determined using equations S3-6 in ESM. The MF was calculated by the ratio of the peak area in the presence of matrix (blank matrix spiked after extraction), to the peak area in the absence of matrix (pure analyte solution). The IS-MF was calculated by division of the MF of the analyte by the MF of the corresponding IS. Thereby, the IS was added as described in the section “Sample preparation.” The RE was determined by calculating the ratio of the peak area extracted with the matrix (blank matrix spiked before extraction) to the peak area in the presence of the matrix (blank matrix spiked after extraction). The IS-RE was calculated by division of the RE of the analyte by the RE of the corresponding IS. The CVs of the MF, the IS-MF, and the IS-RE should be within 15% [19].

The stability of stock solutions in methanol at − 20 °C in amber glass was tested for all analytes over a time of 18 weeks (*n* = 3). Furthermore, benchtop stability (24 h, *n* = 3), one cycle of freeze–thaw stability (*n* = 3), and autosampler stability (24 h, 10 °C, *n* = 3) of processed samples was tested for each sample matrix using QC level 4 (Table 1). Determined QC concentrations should be within ± 15% of the nominal concentration when analyzed after the evaluated storage conditions.

### Proof of concept

As a proof of concept, matching patient samples of plasma, urine, VAMS tips soaked with FPB, and OF sampled by Quantisal of 25 individuals were analyzed. Four patients were prescribed with abemaciclib, anastrozole, exemestane, ribociclib, and tamoxifen, nine patients with letrozole, and eight patients with palbociclib. No patient was prescribed with endoxifen; however, endoxifen was analyzed as tamoxifen metabolite in samples from four patients prescribed with tamoxifen. EDTA-stabilized blood was centrifuged for 5 min at 1645 × *g* to generate plasma. Plasma, urine, VAMS tips, and OF buffer solution were stored at − 20 °C before analysis. Each analytical batch consisted of a blank sample, a zero sample, containing IS but no OET, four QC levels, and the patient samples. If more than 15 patient samples were analyzed in one batch, blank sample, zero sample, and QCs must be reinjected after every 15 patient samples. Medication plans of patients were provided and the assessment of adherence was performed using available reference concentrations for plasma (Table 1). The sampling procedure for VAMS and OF is represented in ESM Figures S1-2.

## Results and discussion

### Method development

Column testing identified a TF Accucore Phenyl-Hexyl column to be the most suited for the current set of analytes. Target analytes were chromatographically separated within 6.5 min using a total run time of 9.1 min (Figure S3, ESM). The TF Hypersil GOLD C18 using mobile phase A (aqueous ammonium formate, 10 mM with formic acid 1%, v/v) and mobile phase B (ACN with formic acid, 0.1%, v/v) showed coelution of abemaciclib and palbociclib as well as coelution of letrozole and anastrozole. The waters AQUITY UPLC BEH C18 column using the mobile phases A (aqueous ammonium formate, 10 mM with formic acid 1%, v/v) and B (ACN with formic acid, 0.1%, v/v) showed general tailing of analytes. As for the Macherey–Nagel HILIC Nucleodur column and Merck SeQuant ZIC HILIC column, peak separation using mobile phase A (aqueous ammonium formate, 200 mM with acetic acid, pH5) and mobile phase B (ACN with formic acid, 0.1%, v/v) was barely possible. Full MS mode showed the lowest detection limits and best peak description. Since this method was developed in the context of adherence monitoring where prescribed medication is known, the exact masses of the positively ionized parent compound in combination with the retention time were considered sufficient for compound identification (ESM Table S1). During the testing of different sample preparations, we found that sample extractions with chlorbutanol resulted in highly variable recoveries for each sample matrix. Hydration of the VAMS tip led to red-colored extracts which needed to be precipitated by a large volume of MeOH or ACN. However, peak areas of OET were not higher compared to sample preparation without hydration of the VAMS tip. For the OF buffer solution, the exact content is not known. Evaporation of OF buffer solution after precipitation resulted in crystalline deposits in the vial which influenced reconstitution of the samples.

### Method validation

Quantification was performed via RRF (ESM Table S1), which was shown to be time-saving since no calibration curves were needed. Furthermore, a stable isotope-labeled IS with identical chemical properties as the targeted analyte may compensate for issues with stability, MF, RE, and ion suppression [31, 32]. However, the purchase of isotope-labeled corresponding IS for each analyte is costly.

Since selectivity testing revealed no interfering signals from endogenous compounds or false positive results in all four matrices, the retention time in combination with the exact ion mass of the compound was considered sufficient for analyte identification. No carry-over greater than 15% of the LLOQ was observed in either matrix after injection of the QC level 4. However, samples following an injection of an even higher concentration should be reanalyzed after one or several wash-out runs with extracted blank. Washing runs before the following sample injection must be carried out until no more analyte carry-over can be observed.

Results for the within- and between-day accuracy and precision are represented in ESM Table S2-5. For plasma as sample matrix, all analytes met the required criteria according to the ICH M10 guideline with the exception of exemestane [20]. For urine as sample matrix, all analytes met the required criteria with the exception of exemestane and letrozole (QC level 1). Consequently, exemestane could only be covered in a qualitative nature in plasma and urine as sample matrix and the LLOQ for letrozole in urine was raised to QC level 2. However, letrozole was still detectable in QC level 1. For VAMS as sample matrix, the required criteria were fulfilled except for endoxifen (QC levels 1 and 2), letrozole (QC level 1), and tamoxifen (QC level 1). However, except for endoxifen (QC level 1), all analytes were detected and allowed for a qualitative adherence assessment over the lower range. The LLOQ in VAMS as sample matrix for letrozole and tamoxifen was raised to QC level 2 and the LLOQ for endoxifen must be raised to QC level 3. Exemestane had a CV of 16% for between-day precision at QC level 4 in VAMS as sample matrix; however, this was considered acceptable. For OF as sample matrix, analytes met the required criteria except for endoxifen, exemestane, and palbociclib at QC level 1; therefore, the LLOQ was raised to QC level 2. However, the qualitative assessment was still possible for those three analytes at QC level 1. Furthermore, letrozole and its corresponding IS could not be detected in this sample matrix possibly due to matrix interference by ion suppression; therefore, the adherence assessment for letrozole in OF was not possible. Chromatograms of LLOQs in the different matrices are represented in ESM Figure S4-S7.

Determined MF, IS-MF, and IS-RE values, as well as corresponding CVs for the analytes, are represented in ESM Tables S6-8. In extracted plasma samples, IS-MF of analytes varied between 98% (palbociclib QC level 2) and 103% (letrozole QC level 2 and ribociclib QC level 2 and 4) with CVs within 3%. In extracted urine samples, IS-MF varied between 98% (endoxifen QC level 2) and 106% (exemestane QC level 2) with CVs within 10% except for letrozole with an IS-MF of 130% (QC level 2) and 124% (QC level 4) and CVs of 31% for QC level 2 and 21% for QC level 4. In extracted VAMS tip samples, IS-MF varied between 96% (exemestane QC level 2) and 108% (endoxifen QC level 2) with CVs within 4%. However, IS-MF was only determined for a hematocrit level of 40%; therefore, higher deviations cannot be excluded for lower or higher hematocrit levels, although VAMS is claimed to be independent of the hematocrit level [33]. In extracted OF samples, IS-MF varied between 80% (anastrozole QC level 2) and 110% (palbociclib QC level 2). Although IS-MF was higher than for the other sample matrices, it was found to be reproducible with CVs within 12%. As additional information, MF is represented in ESM Table S7. IS-RE are represented in ESM Table S8. For plasma as sample matrix, IS-RE varied between 87% (tamoxifen QC level 4) and 102% (endoxifen QC level 4) with CV within 5% and comparable IS-RE nominal values between QC levels. For urine as sample matrix, IS-RE varied between 87% (tamoxifen QC level 4) and 106% (letrozole QC level 2) with CVs within 8% except for letrozole (23% for QC level 2). Nominal values of IS-RE are comparable between QC levels in urine as sample matrix. For VAMS as sample matrix, IS-RE varied between 56% (ribociclib QC level 4) and 103% (letrozole QC level 2). Although IS-RE was quite low for some analytes, CVs were within 15% and nominal values of IS-RE were comparable between QC levels. For OF as sample matrix, IS-RE varied between 22% (tamoxifen QC level 2) and 101% (anastrozole QC level 4). Again, IS-RE was quite low for some analytes; however, CVs were within 13% and nominal values of IS-RE were comparable between QC levels. In general, extraction procedures for plasma and urine yielded high IS-RE values, for VAMS an OF some analytes showed rather low IS-RE values, however they were reproducible and therefore deemed adequate.

The stability of analytes in methanolic stock solutions was tested and all analytes and IS were found to be stable over at least 18 weeks at − 20 °C in amber glass vials since no degradation exceeding 10% was detected. Results of benchtop stability in all four matrices are given in ESM Table S9. No analyte showed a degradation over 15% of the nominal concentration for the tested QC level 4 after storage for 24 h at room temperature (24 °C). Furthermore, CVs were within 15%. Results for one cycle of freeze–thaw stability for all four matrices are given in ESM Table S10. Again, no degradation over 15% of the nominal concentration of the QC level 4 could be observed and CVs were within 15%. Testing for one freeze–thaw cycle was considered sufficient, since adherence monitoring is either performed immediately after samples arrive at the laboratory or samples are analyzed in a batch at certain intervals. Testing multiple freeze–thaw cycles would not correspond to reality. Results for autosampler stability at 10 °C for 24 h of the four different extracted sample matrices are given in ESM Table S11. No degradation over 15% of the nominal concentration of the QC level 4 could be observed with CVs within 15%. Overall stability testing revealed consistent results for the different storage conditions and sample matrices. However, the stability of analytes exceeding the tested conditions cannot be guaranteed. Furthermore, it must be considered that spiked samples may not always display the same stability profile as actual samples [25].

Only for plasma as sample matrix, reference concentrations for steady state, minimal or trough, and maximal concentrations were available in the literature. Therefore, ranges for quantifications were considered for these data for all four matrices (see Table 1). The present methods allow quantification of abemaciclib, and palbociclib from the reported minimal to maximal concentration in all four sample matrices. For anastrozole, the trough concentration as well as the steady-state concentration was covered quantitatively in all four matrices; however, no maximal concentration was available. For ribociclib, the maximal concentration was above the validated range; however, for the purpose of adherence monitoring, an exact quantification in the upper reference range is not necessary. More important is the quantification of the trough concentration, and this could be covered in all four sample matrices. Reported minimal to maximal concentration for letrozole could be covered quantitatively in plasma. In VAMS and urine, only higher concentrations could be covered quantitatively and letrozole was not detectable in OF. For tamoxifen, the trough concentration could be covered quantitatively for plasma, OF, and urine; however, not for VAMS as sample matrix. The steady-state reference range of tamoxifen was above the validated range, yet this did not impact the adherence assessment. The current procedure cannot only be used to monitor tamoxifen, but also endoxifen in human biosamples. Endoxifen is a secondary tamoxifen metabolite resulting from cytochrome P450 (CYP) 2D6-dependent biotransformation of the primary tamoxifen metabolite [34]. CYP2D6 is widely known for its inter-individual variation in the metabolic capacity and poor metabolizers of tamoxifen were shown to have lower levels of endoxifen and poorer clinical outcomes as compared to extensive metabolizers [35]. Therefore, endoxifen is considered to represent the major active metabolite of tamoxifen and is currently investigated as an independent anti-cancer drug [34, 35]. In the current study, its steady-state concentrations in patients treated with tamoxifen could be covered quantitatively except for VAMS as sample matrix. Trough concentrations of exemestane could not be covered quantitatively in any of the four matrices. Furthermore, qualitative detection of all OETs was possible at least down to QC level 1 in all tested sample matrices, except for letrozole in OF. However, including further metabolites for the purpose of adherence monitoring is not necessary. The quantification of the parent compound in patient samples (particularly OF, whole blood, plasma) and comparison to trough concentration is sufficient.

### Proof of concept

As a proof of concept, 25 authentic matching plasma, urine, and VAMS tips soaked with FPB and OF samples were analyzed. For patients number 2 and 16, no urine was available, and for patient number 9, no VAMS tip soaked with FPB was available for the analysis. Four patients were prescribed amongst others abemaciclib, anastrozole, exemestane, ribociclib, and tamoxifen, nine patients were prescribed amongst others letrozole, and eight patients were prescribed amongst others palbociclib. Unfortunately, no patient was prescribed endoxifen; however, endoxifen was monitored as a metabolite of tamoxifen for four intakes. Most OETs are taken daily; however, according to the recommended dosing regimens, palbociclib and ribociclib are orally administered for 21 days in a row, followed by 7 days off treatment (28-day cycle). Only two of the eight monitored palbociclib patients were taking palbociclib actively; however, all four ribociclib patients were taking ribociclib actively.

Table 2 shows the quantitative results in the four sample matrixes as well as the assessment of adherence. Cut-off concentrations for adherence assessment in plasma were based on trough concentration of OET available in literature, except for endoxifen where only steady-state concentrations were available (Table 1). However, these cut-off concentrations need to be clinically validated against measured drug concentrations in patients known to be adherent. Additionally, reference concentrations for urine, VAMS, and OF need to be established. Therefore, only qualitative adherence assessment was possible using those three sample matrices.

**Table 2:** Quantification of oral endocrine therapies (OET) (ng mL^−1^) in plasma, urine, finger prick blood sampled by volumetric absorptive microsampling (VAMS), and oral fluid (OF) and assessment of adherence using available plasma reference ranges (Table 1). Prescribed medication and mode of intake provided by medication plans. Mode of intake described as number of tablets taken in the morning-noon-evening. (# device not completely soaked, ↑ classified as adherent, ↓ classified as nonadherent, *conc.* concentration, *metab.* metabolite, *n.a.* not available, *n.d.* not detectable)

Patient	OET	Mode of intake	Hours since last intake	Conc. in plasma, ng mL^−1^	Conc. in urine, ng mL^−1^	Conc. in VAMS ng mL^−1^	Conc. in OF ng mL^−1^
1	Letrozole 2.5 mg Palbociclib 125 mg	0–0-1 0–0-1	17 17	101*↑* > 100*↑*	< 50*↑* > 100*↑*	< 150*↑* > 100*↑*	n.d.*↓* > 100*↑*
2	Letrozole 2.5 mg Palbociclib 100 mg	0–1-0 1–0-0	23 7 days	< 50*↓* < 10*↓*	n.a n.a	< 150*↑* < 10*↑*	n.d.*↓* < 30*↑*
3	Exemestane 25 mg	1–0-0	2	> 100*↑*	28*↑*	18*↑*	n.d.*↓*
4	Ribociclib 200 mg	0–0-1	10	681*↑*	> 100*↑*	558*↑*	# > 1000*↑*
5	Tamoxifen 20 mg Endoxifen as metab	0–0-1	16	42*↑* 1*↓*	n.d.*↓* n.d.*↓*	43*↑* n.d.*↓*	< 10*↑* n.d.*↓*
6	Palbociclib 125 mg Anastrozole 1 mg	1–0-0 0–0-1	7 days 13	< 10*↓* 35*↑*	> 100*↑* 38*↑*	13*↑* 54*↑*	< 30*↑* 20*↑*
7	Palbociclib 125 mg Letrozole 2.5 mg	0–1-0 1–0-0	22 2	> 100*↑* 137*↑*	> 100*↑* 128*↑*	#n.a #n.a	#98*↑* n.d.*↓*
8	Exemestane 25 mg	1–0-0	3	47*↑*	40*↑*	99*↑*	n.d.*↓*
9	Palbociclib 125 mg	1–0-0	8 days	36*↓*	> 100*↑*	n.a	< 30*↑*
10	Palbociclib 100 mg Letrozole 2.5 mg	1–0-0 0–1-0	7 days 20	13*↓* 50*↓*	> 100*↑* n.d.*↓*	15*↑* < 150*↑*	n.d.*↓* n.d.*↓*
11	Letrozole 2.5 mg Abemaciclib 150 mg	0–0-1 1–0-1	11 11	159*↑* 165*↓*	> 500*↑* 306*↑*	# n.a # n.a	n.d.*↓* 95*↑*
12	Exemestane 25 mg Tamoxifen 20 mg Endoxifen as metab	0–0-1 0–0-1 n.a	12 12 n.a	27*↑* > 100*↑* 18*↑*	10*↑* < 10*↑* 9*↑*	< 10*↑* 94*↑* < 50*↑*	n.d.*↓* < 10*↑* n.d.*↓*
13	Ribociclib 200 mg	0–0-1	35	> 1000*↑*	> 100*↑*	> 1000*↑*	390*↑*
14	Ribociclib 600 mg Letrozole 2.5 mg	0–0-1 0–0-1	18 18	970*↑* < 50*↓*	> 100*↑* 94*↑*	> 1000*↑* < 150*↑*	386*↑* n.d.*↓*
15	Abemaciclib 150 mg	1–0-1	2	116*↓*	> 450*↑*	147*↑*	153*↑*
16	Palbociclib 125 mg Anastrozole 1 mg	1–0-0 1–0-0	7 days 3	13*↓* 36*↑*	n.a n.a	< 10*↑* 15*↑*	< 30*↑* < 10*↑*
17	Tamoxifen 20 mg Endoxifen as metab	0–0-1 n.a	16 n.a	> 100*↑* 7*↓*	n.d.*↓* n.d.*↓*	61*↑* < 50*↑*	< 10*↑* n.d.*↓*
18	Tamoxifen 20 mg Endoxifen as metab	0–0-1 n.a	10 n.a	91*↑* 5*↓*	1 < 10 n.d.*↓*	68*↑* < 50*↑*	12*↑* n.d.*↓*
19	Exemestane 25 mg	0–0-1	12	22*↑*	4 < 10*↑*	23*↑*	n.d.*↓*
20	Abemaciclib 150 mg Letrozole 2.5 mg	1–0-1 0–0-1	12 12	113*↓* 84*↓*	> 450*↑* < 150*↑*	130*↑* < 150*↑*	90*↑* n.d.*↓*
21	Palbociclib 125 mg Letrozole 2.5 mg	0–0-1 0–0-1	8 days 16	< 10*↓* 162*↑*	> 100*↑* 89*↑*	< 10*↑* 151*↑*	n.d.*↓* n.a
22	Anastrozole 1 mg	0–0-1	14	< 10*↓*	< 10*↑*	< 10*↑*	n.d.*↓*
23	Ribociclib 600 mg	1–0-0	14	541*↑*	> 100*↑*	> 1000*↑*	571*↑*
24	Anastrozole 1 mg	0–0-1	14	< 10*↓*	12*↑*	< 10*↑*	n.d.*↓*
25	Abemaciclib 150 mg Letrozole 2.5 mg	1–0-1 0–0-1	3 14	363*↑* 97*↑*	> 450*↑* 116*↑*	303*↑* < 150*↑*	116*↑* n.d.*↓*

Determined OET concentrations differed between the sample matrices. The highest concentrations were determined in urine as sample matrix, except for endoxifen, exemestane, tamoxifen, and partially letrozole. Even though abemaciclib (81%), palbociclib (74%), and ribociclib (69%) are reported to be mainly excreted via feces, high concentrations could be determined in urine [18]. It should be noted that urinary concentrations are affected by urine flow, frequency of bladder emptying, and urinary pH [7]. Therefore, the benefit of urinary drug concentrations is controversial and challenging for interpretation [7]. We propose to evaluate the determined urinary concentrations on a qualitative basis. If OET can be detected, the patient is considered adherent. Quantitative method validation in urine as sample matrix is nevertheless useful to be aware of the limitations of the method.

Quantification results in FPB sampled by VAMS and plasma cannot be used interchangeably. For abemaciclib, two out of three concentrations were determined higher in FPB than in plasma. For anastrozole, one concentration was determined higher in FPB and one concentration inferior in FPB compared to plasma. For two patients, the determined concentration was below the LLOQ in both matrices. For exemestane, half of the determined concentrations in FPB were higher, and half inferior to plasma concentrations. Letrozole FPB concentrations were in six patients inferior to the LLOQ and for one patient the letrozole concentration was inferior in FPB than in plasma. Palbociclib was determined three times below the LLOQ and once above the upper ULOQ. For patient number 10, the determined concentrations were comparable in FPB and plasma. Ribociclib was determined in two out of four patients at a higher concentration in FPB than plasma. For one patient, the FPB concentration was inferior to the plasma concentration, and for one patient, the determined concentration in both matrices was above the ULOQ. Tamoxifen concentrations in FPB were determined inferior in three out of four cases and for one patient concentrations were comparable between FPB and plasma. Endoxifen, the active metabolite of tamoxifen, was detected in FPB in three out of four patients; however, concentrations were below the LLOQ. FPB consists of capillary whole blood where total drug levels are measured, whereas plasma measurements do not include intracellular drug levels [36]. Lipophilic drugs can cross the red blood cell (RBC) lipid membrane by diffusion, while hydrophilic drugs may enter, e.g., due to aqueous channels or active transporter systems. Once the drug is in the RBC cytoplasm, it can bind to enzymes and/or proteins. Tacrolimus, used in breast cancer therapy, e.g., exhibits increased affinity towards RBC due to its interaction with tacrolimus-binding proteins found within RBC [37, 38]. Therefore, amongst other factors, it is crucial to consider the distinct nature of plasma and whole blood.

Amongst others, therefore, the consideration of the different natures of plasma and whole blood is important.

Since some drugs can be distributed into red blood cells, e.g., lipophilic drugs can cross the red blood cell lipid by diffusion, membrane consideration of the different matrices is important [39].

To the best of our knowledge, no information on the detectability of OET in OF is available. Abemaciclib was detectable in OF in all four sampled patients and three concentrations were inferior to plasma concentrations. Anastrozole could be detected in two out of four patients’ OF samples with inferior concentrations than in plasma. Exemestane and letrozole could not be detected in any of the patients’ OF samples. For letrozole, problems with detectability were already shown during method validation; however, exemestane could not be detected in any of the four patients even though all four patients were assessed as adherent using plasma as sample matrix. Palbociclib could be detected in six out of eight OF samples, comparison of OF concentrations to plasma concentration is not possible since determined concentrations were outside the validated concentration range or in the case of patient number 4 the OF sampling device was not completely soaked with OF which is influencing the determined concentration. Ribociclib was detectable in all four OF patient samples with half of the determined concentrations inferior and half superior to plasma concentrations. Tamoxifen could be detected in all four OF patient samples; however, in three cases, the determined concentration was below the LLOQ. Nevertheless, all four determined concentrations in OF were inferior to determined concentrations in plasma. Endoxifen as the active metabolite of tamoxifen could not be detected in any of the patient OF samples. OF is considered to reflect the free fraction of circulation drug; in other words, protein-bound drugs may not be detected in OF [7]. Furthermore, the salivary flow, the pH of OF, the pK_a_, molecular weight, and lipid solubility of a drug have an influence on its detectability in OF [7]. Moreover, contaminations of the oral cavity with recently ingested OET can result in high concentrations of OF.

Quantification results in FPB or OF and plasma cannot be used interchangeably. It is necessary to establish reference ranges by sampling many known adherent patients at the trough concentration in a controlled study. Nevertheless, the current results demonstrate that VAMS and OF are a suitable tool for adherence monitoring of selected OET.

Using plasma as sample matrix, patients 2, 6, 9, 10, 16, and 21 were assessed as nonadherent to palbociclib due to concentrations below the trough concentration. However, all these patients were in the off-treatment part of the 28-day intake cycle and thus out of a steady-state kinetic. Patients number 2, 10, 14, and 17 were assessed as nonadherent to letrozole, patients 11, 15, and 17 were assessed as nonadherent to abemaciclib, and patients number 22 and 24 were assessed as nonadherent to anastrozole. However, in all plasma samples, the prescribed OET was still detectable, even though concentrations were below the trough concentrations. This could indicate that trough concentrations need to be evaluated clinically for these OETs. Patients number 5, 12, 17, and 18 were assessed as adherent to tamoxifen; however, three out of four patients showed an endoxifen concentration below the available steady-state concentration, indicating that an adaption of this concentration should be taken into consideration.

Using urine as sample matrix, only patients 5, 10, 17, and 18 were assessed as non- or partially adherent. Patient number 10 was assessed as partially nonadherent towards letrozole which is in alliance with the determined plasma concentration. For patients 5, 17, and 18, tamoxifen and its metabolite endoxifen could not be detected in urine; however, the patients were assessed as adherend regarding the tamoxifen concentration in plasma.

Overall, a high rate of adherence towards OET was notable. However, adherence was assessed for only a small collective of patients, and background information on the duration of the ongoing treatment is missing.

### Limitations

Trough concentrations can be used as cut-off concentrations for adherence assessment. However, reference trough concentrations are only available for plasma. Thus, there is a need to establish reference trough concentrations for urine, VAMS, and OF. Additionally, these reference ranges need to be clinically validated for all four sample matrices. This analytical method was therefore developed and validated to detect and quantify OET in the different matrices and target values can now be determined or correlations between matrices can be established. Furthermore, the direct adherence monitoring by LC-HRMS cannot exclude white coat adherence, where patients only take medication in anticipation of a monitoring event. Moreover, single skip days of medication intake will probably not influence the concentration in the different matrices to notice a difference in concentration since OET have rather long half-lives. However, physicians report that in the case of OET, patients are often fully adherent or completely nonadherent.

Some OETs are barely excreted via urine or are strongly metabolized before urinary excretion which can influence the adherence assessment via detectability of the parent compound. Furthermore, the general detectability of OET or their metabolites in OF was so far not reported in literature. These circumstances could lead to an underestimation of adherence. Therefore, an additional screening procedure for the presence of metabolites in urine or OF might be reasonable.

VAMS is used to sample FPB and is therefore suitable for at-home sampling. However, patients need to be trained rigorously to avoid false results due to sampling errors. For some patients, FPB sampling might be difficult. This was observed, e.g., for patients number 7 and 11. VAMS tips could not be completely filled with FPB due to circulatory pathologies. Furthermore, FPB is whole (capillary) blood and determined concentrations cannot always be converted into plasma concentrations [11, 40]. A further challenge of whole blood sampling is the influence of the hematocrit level. VAMS is claimed to allow for sampling of an accurate volume independent of the hematocrit; however, MF could be influenced by the hematocrit level. Moreover, in the case of home sampling, where the device is sent via mail delivery to the laboratory, the influence of, e.g., temperature and humidity during transport should be investigated in future studies.

OF is also suitable for at-home sampling, requiring the same training of patients and investigations of the influence of mail delivery as for VAMS. Furthermore, dry mouth syndrome can cause difficulty in the OF sampling procedure. This was the case for patient number 4. In contrast to VAMS, where the laboratory staff can visually assess if the VAMS tip is completely soaked or not, for OF sampling with Quantisal, this is not possible. If the cotton swab is only partly soaked with OF, the color indicator on the tips will nevertheless turn blue since the cotton swab is filled with buffer solution. So, patients need to actively report any sampling difficulties.

## Conclusion

Analytical procedures for the direct adherence monitoring of OET in four different matrices were successfully developed and validated. A proof of concept where 41 OET concentrations in 25 different patients and four sample matrices were determined was carried out. Each investigated matrix has its own benefits and challenges, e.g., trough concentrations were only available for plasma as sample matrix and urine, VAMS, and OF allow noninvasive collection, suitable for at-home sampling. Furthermore, reference ranges in FPB sampled by VAMS and in OF are necessary for adherence assessment and these reference ranges need to be clinically validated. The presented method also allows future studies on the pharmacokinetics of OET, e.g., in which extent is the free part of tamoxifen transferred from the blood into OF and how is its pH dependence or how is the transfer of particular drugs physiologically controlled into OF.

### Supplementary Information

Below is the link to the electronic supplementary material.

Supplementary file1 (PDF 1475 KB)

## Data Availability

The datasets generated and analyzed during the current study are available from the corresponding author on reasonable request.
