# Supplementary material for: Towards clinical adherence monitoring of oral endocrine breast cancer therapies by LC-HRMS—method development, validation, comparison of four sample matrices, and proof of concept
Source: Anal Bioanal Chem. 2024 Mar 15;416(12):2969–81. doi: 10.1007/s00216-024-05244-6 (PMC11045636; doi:10.1007/s00216-024-05244-6)
Supplement: Supplementary file 1 — Supplementary file1 (PDF 1475 KB) [file 216_2024_5244_MOESM1_ESM.pdf]

Electronic supplementary material

Towards clinical adherence monitoring of oral endocrine breast cancer therapies by LC-HRMS - Method development, validation, comparison of four sample matrices, and proof of concept

Cathy M. Jacobs[1], Julia C. Radosa[2], Lea Wagmann[1], Julia S. M. Zimmermann[2], Askin C. Kaya[2], Aylin Aygün[2], Tatjana Edel[2], Lisa Stotz[2], Mohamed Ismaeil[2], Erich-Franz Solomayer[2], Markus R. Meyer[1]

[1]Department of Experimental and Clinical Toxicology, Saarland University, Homburg, Germany

[2]Department of Gynaecology, Obstetrics and Reproductive Medicine, Saarland University Hospital, Homburg, Saarland, Germany

Table S1: exact ion masses $[M+H]^+$ of analytes and isotope labeled internal standards, corresponding retention time (RT), and relative response factor (RRF) in plasma, urine, volumetric absorptive microsampling (VAMS), and oral fluid (OF) used for quantification. (n.a.: not available)

**Compound**	$[M+H]^+$	**RT**	**RRF plasma**	**RRF urine**	**RRF VAMS**	**RRF OF**
Abemaciclib	507.2791	2.7	1.6	1.6	1.5	0.60
Abemaciclib-d_8	515.3293	2.7	n.a.	n.a.	n.a.	n.a.
Anastrozole	294.1713	5.2	1.1	1.1	1.2	1.25
Anastrozole-$^{13}C_4$	298.1847	5.2	n.a.	n.a.	n.a.	n.a.
Endoxifen	374.2115	5.7	0.55	0.45	0.35	0.35
Endoxifen-d_5	379.2428	5.7	n.a.	n.a.	n.a.	n.a.
Exemestane	297.1849	6.0	1.2	1.2	0.80	1.0
Exemestane-$^{13}C,d_3$	301.2071	6.0	n.a.	n.a.	n.a.	n.a.
Letrozole	286.1087	5.0	1.0	1.2	0.95	n.a.
Letrozole-$^{13}C_2,^{15}N_2$	290.1095	5.0	n.a.	n.a.	n.a.	n.a.
Palbociclib	448.2455	3.0	0.95	0.95	0.70	1.0
Palbociclib-d_8	456.2958	3.0	n.a.	n.a.	n.a.	n.a.
Ribociclib	435.2615	2.1	1.1	1.1	0.80	1.1
Ribociclib-d_6	441.2992	2.1	n.a.	n.a.	n.a.	n.a.
Tamoxifen	372.2322	6.4	1.1	1.0	1.1	0.35
Tamoxifen-d_5	377.2636	6.4	n.a.	n.a.	n.a.	n.a.

Table S2: Within- and between-day accuracy and precision of the quality control (QC) 1-4 (n=5 at three different days) in plasma.

**Analyte**	**Relative mean concentration (accuracy), %; CV (precision), %**							
	**Within-day**				**Between-day**			
	**QC1**	**QC 2**	**QC 3**	**QC 4**	**QC 1**	**QC 2**	**QC 3**	**QC 4**
Abemaciclib	94; 6	95; 2	100; 5	108; 2	101; 5	100; 5	101; 2	102; 9
Anastrozole	96; 4	103; 2	101; 2	102; 2	100; 4	108; 5	102; 2	98; 7
Endoxifen	82; 7	94; 2	105; 4	105; 2	81; 2	98; 4	107; 2	98; 12
Exemestane	165; 25	143; 6	111; 4	110; 3	162; 10	150; 13	119; 6	103;20
Letrozole	92; 9	94; 3	88; 4	106; 2	99; 7	99; 5	89; 2	99; 10
Palbociclib	98; 8	101; 3	108; 4	113; 3	91; 12	98; 5	110; 2	105; 9
Ribociclib	96; 9	91; 2	91; 5	103; 2	104; 8	99; 7	99; 8	97; 8
Tamoxifen	110; 7	109; 1	110; 4	115; 9	117; 6	114; 7	108; 3	103; 12

Table S3: Within- and between-day accuracy and precision of the quality control (QC) 1-4 (n=5 at three different days) in urine. (n.a.: not available)

**Analyte**	**Relative mean concentration (accuracy), %; CV (precision), %**							
	**Within-day**				**Between-day**			
	**QC1**	**QC 2**	**QC 3**	**QC 4**	**QC 1**	**QC 2**	**QC 3**	**QC 4**
Abemaciclib	113; 3	108; 3	108; 3	109;2	109; 3	103; 5	108; 1	108; 5
Anastrozole	106; 1	115; 3	110; 1	104; 2	104; 1	110; 4	107; 2	101; 4
Endoxifen	92; 6	106; 4	115; 4	109; 1	86; 6	105; 5	114; 1	108; 6
Exemestane	111; 24	96; 13	118; 27	112; 20	96; 25	109; 10	103;15	114;7
Letrozole	40;28	90; 8	92; 6	104; 2	47;15	91; 3	94; 7	112; 5
Palbociclib	90; 10	90; 7	109; 5	94; 4	96; 10	100;10	109; 2	102; 7
Ribociclib	107; 3	102; 5	111; 3	100; 2	106; 1	98; 5	107; 8	99; 4
Tamoxifen	115; 2	100; 1	99; 2	93; 1	113; 2	99; 3	100; 2	91; 6

Table S4: Within- and between-day accuracy and precision of the quality control (QC) 1-4 (n=5 at three different days) in volumetric absorptive microsampling. (n.a.: not available)

Analyte	Relative mean concentration (accuracy), %; CV (precision), %							
	Within-day				Between-day			
	**QC1**	**QC 2**	**QC 3**	**QC 4**	**QC 1**	**QC 2**	**QC 3**	**QC 4**
Abemaciclib	97; 3	97;6	94; 3	106; 5	97; 0	96; 1	93; 2	105; 5
Anastrozole	114; 3	114; 4	103; 2	100;5	114; 1	112; 2	101; 5	101; 6
Endoxifen	n.a.	50; 8	100; 4	112; 6	n.a.	58; 27	101; 1	107; 5
Exemestane	92; 2	110; 6	114; 2	113; 3	91; 7	103; 10	107; 13	105; 16
Letrozole	75; 6	97; 9	89; 2	102; 4	63; 20	95; 4	86; 6	102; 7
Palbociclib	86; 3	96; 6	99; 3	105; 9	88; 6	94; 4	101; 4	105; 1
Ribociclib	110; 2	93; 6	96; 3	97; 6	112; 5	94; 3	99; 2	97; 1
Tamoxifen	134; 2	103; 4	96; 2	93; 5	131; 2	101,2	91,6	90,9

Table S5: Within- and between-day accuracy and precision of the quality control (QC) 1-4 (n=5 at three different days) in OF. (n.a.: not available)

Analyte	Relative mean concentration (accuracy), %; CV (precision), %							
	Within-day				Between-day			
	**QC1**	**QC 2**	**QC 3**	**QC 4**	**QC 1**	**QC 2**	**QC 3**	**QC 4**
Abemaciclib	100; 5	111; 9	97; 5	96; 3	102; 12	102; 8	98; 5	92; 9
Anastrozole	83; 10	111; 4	98; 3	104; 3	81; 5	110; 1	98; 1	109; 4
Endoxifen	96; 3	107; 3	108; 3	99; 2	61; 75	104; 10	104; 5	106; 12
Exemestane	67; 9	103; 3	99; 4	102; 5	60; 30	95; 9	98; 6	108; 7
Letrozole	n.a.	n.a.	n.a.	n.a.	n.a.	n.a.	n.a.	n.a.
Palbociclib	150; 6	107; 6	106; 3	90; 3	171; 22	111;5	105; 3	91; 0
Ribociclib	90; 2	94; 2	97; 3	95; 2	90; 0	92; 2	97; 1	92; 5
Tamoxifen	112; 1	101; 2	112; 3	87; 2	113; 3	111; 11	97; 15	89; 3

Table S6: internal standard (IS) normalized matrix factor (MF) (n=6) of analytes in plasma, urine, volumetric absorptive microsampling (VAMS), and oral fluid (OF) worked up samples. (QC: quality control; CV: coefficient of variation; n.a.: not available)

**Analyte**	**IS normalized MF, %; CV, %**							
	**Plasma**		**Urine**		**VAMS**		**OF**	
	**QC2**	**QC4**	**QC2**	**QC4**	**QC2**	**QC4**	**QC2**	**QC4**
Abemaciclib	101; 3	101; 3	102; 4	101; 4	100; 2	100; 1	88; 4	94; 4
Anastrozole	101; 1	101; 0	102; 2	103; 2	100; 1	100; 1	80; 3	97; 4
Endoxifen	101; 1	101; 1	98; 1	101; 1	108; 3	100;3	94; 2	99; 2
Exemestane	101; 2	102; 2	106; 10	105; 10	96; 4	100; 1	89; 12	102; 4
Letrozole	103; 2	102; 2	130; 31	124; 21	98; 3	102; 2	n.a.	n.a.
Palbociclib	98; 3	100; 3	101; 2	103; 3	102; 4	100; 2	110; 6	104; 4
Ribociclib	103; 3	103; 2	104; 4	105; 3	100; 1	100; 1	91; 2	95; 5
Tamoxifen	100; 2	101; 1	102; 1	101; 1	102; 1	102; 1	97; 4	102; 4

Table S7: matrix factor (MF) (n=6) of analytes and internal standards in plasma, urine, volumetric absorptive microsampling (VAMS), and oral fluid (OF) worked up samples. (QC: quality control; CV: coefficient of variation; N.A.: not available)

**Analyte**	**MF, %; CV, %**							
	**Plasma**		**Urine**		**VAMS**		**OF**	
	**QC2**	**QC4**	**QC2**	**QC4**	**QC2**	**QC4**	**QC2**	**QC4**
Abemaciclib	103; 7	97;4	85; 23	80; 23	90; 5	91; 8	68; 9	47; 21
Abemaciclib-d_8	102; 7	96; 4	84; 25	79; 23	90; 6	91; 9	76; 5	50; 26
Anastrozole	118; 16	111; 7	125; 10	121; 7	102; 2	99; 1	105; 13	81; 4
Anastrozole-$^{13}C_4$	117; 16	109; 7	123; 11	118; 7	102; 1	98; 1	131; 13	83; 6
Endoxifen	93; 5	94; 6	83; 6	90; 8	101; 3	91; 2	120; 15	80; 5
Endoxifen-d_5	93;5	93;6	85; 6	89; 7	93; 1	91; 2	124; 16	81; 5
Exemestane	135; 8	120; 4	117; 17	103; 14	103; 3	100; 2	103; 19	86; 12
Exemestane-$^{13}C,d_3$	134; 8	118; 4	113; 22	99; 20	106; 2	100; 2	116; 17	84; 11
Letrozole	104; 4	101; 5	76; 9	78; 10	105; 4	108; 2	N.A.	N.A.
Letrozole-$^{13}C_2,^{15}N_2$	102; 5	99; 6	56; 37	66; 23	107; 3	106; 3	N.A.	N.A.
Palbociclib	109; 10	107; 6	118; 12	108; 29	109;5	107; 2	107; 16	58; 9
Palbociclib-d_8	111; 12	108; 8	118; 13	116; 14	108; 3	107; 3	98; 13	56; 9
Ribociclib	115; 4	110; 7	37; 38	41; 43	110; 2	102; 4	187; 17	114; 10
Ribociclib-d_6	111; 6	107; 7	36; 41	39; 41	109; 2	111; 13	205; 15	120; 12
Tamoxifen	86; 5	86; 2	87; 4	88; 5	92; 1	91; 3	106; 16	70; 4
Tamoxifen-d_5	86; 4	85; 2	85; 5	87; 7	91; 1	89; 3	110; 13	68; 3

Table S8: Internal standard (IS) normalized recovery (RE) (n=6) of plasma, urine, volumetric absorptive microsampling (VAMS), and oral fluid (OF) worked up samples. (QC: quality control; CV: coefficient of variation; n.a.: not available)

**Analyte**	**IS normalized RE, %; CV, %**							
	**Plasma**		**Urine**		**VAMS**		**OF**	
	**QC2**	**QC4**	**QC2**	**QC4**	**QC2**	**QC4**	**QC2**	**QC4**
Abemaciclib	92; 2	89; 4	93; 2	91; 3	73; 8	79; 7	31; 3	35; 8
Anastrozole	99;3	94; 4	98; 1	90; 3	102; 9	99; 10	92; 6	101;7
Endoxifen	98; 2	102; 4	97; 1	100; 2	n.a.	50; 15	43; 5	53; 10
Exemestane	100; 3	95; 3	103; 5	99; 8	61; 15	69; 14	87; 11	92; 8
Letrozole	98; 4	90; 4	106; 23	91; 7	103; 8	100; 11	N.A.	N.A.
Palbociclib	100; 2	92; 5	98; 3	91; 7	61; 15	59; 14	68; 4	71; 5
Ribociclib	99; 3	97; 3	99; 4	94; 5	59; 13	56; 15	75; 3	84; 8
Tamoxifen	98; 3	87; 4	98; 1	87; 2	83; 9	80; 9	22; 10	27; 13

Table S9: Benchtop for 24 h at 24°C (n=3) of plasma, urine, volumetric absorptive microsampling (VAMS), and oral fluid (OF) worked up samples. (QC: quality control; CV: coefficient of variation; n.a.: not available)

**Analyte**	**Relative mean concentration, %; CV, %**			
	**Plasma**	**Urine**	**VAMS**	**OF**
	**QC4**	**QC4**	**QC4**	**QC4**
Abemaciclib	104; 6	92; 3	101; 3	107; 1
Anastrozole	102; 6	101; 1	112; 3	109; 4
Endoxifen	115; 7	115; 2	115; 6	115; 4
Exemestane	88; 8	88; 2	105; 5	101; 4
Letrozole	97; 7	91; 2	114; 4	n.a.
Palbociclib	97; 7	115; 2	102; 3	88; 1
Ribociclib	92; 8	99; 5	91; 4	88; 1
Tamoxifen	98; 7	109; 3	93; 3	88; 2

Table S10: Freeze thaw for one cycle at -20°C (n=3) of plasma, urine, volumetric absorptive microsampling (VAMS), and oral fluid (OF) worked up samples. (QC: quality control; CV: coefficient of variation; n.a.: not available)

**Analyte**	**Relative mean concentration, %; CV, %**			
	**Plasma**	**Urine**	**VAMS**	**OF**
	**QC4**	**QC4**	**QC4**	**QC4**
Abemaciclib	104; 3	92; 2	101; 2	107; 2
Anastrozole	98; 1	105; 2	110; 3	105; 3
Endoxifen	93; 2	115; 4	114; 2	115; 0
Exemestane	114; 2	90; 4	113; 1	87; 0
Letrozole	92; 4	94; 2	115; 3	n.a.
Palbociclib	108; 2	114; 2	101; 5	85; 1
Ribociclib	99; 2	98; 0	90; 2	88; 1
Tamoxifen	100; 1	103; 1	108; 3	85; 1

Table S11: Autosampler stability for 24 h at 10°C (n=3) of plasma, urine, volumetric absorptive microsampling (VAMS), and oral fluid (OF) worked up samples. (QC: quality control; CV: coefficient of variation; n.a.: not available)

**Analyte**	**Relative mean concentration, %; CV, %**			
	**Plasma**	**Urine**	**VAMS**	**OF**
	**QC4**	**QC4**	**QC4**	**QC4**
Abemaciclib	94; 2	91; 2	106; 5	92; 7
Anastrozole	94; 4	106; 1	106; 5	112; 2
Endoxifen	88; 1	115; 3	105; 5	100; 5
Exemestane	85; 2	94; 3	115; 6	114; 4
Letrozole	92; 3	89; 3	106; 7	n.a.
Palbociclib	95; 4	114; 1	99; 8	94; 6
Ribociclib	88; 6	99; 2	96; 8	95; 4
Tamoxifen	95; 2	103; 1	94; 7	90; 3

$C_A = \frac{area_A}{area_{IS}} \frac{C_{IS}}{RRF}$ (Equation S1)

Equation S1: Calculation of the concentration (C) of an analyte (A) using relative response factor (RRF) of the corresponding isotope labeled Internal standard (IS)

$RRF = \frac{area_A}{area_{IS}} \frac{C_{IS}}{C_A}$ (Equation S2)

Equation S2: Determination of relative response factor (RRF) using peak areas of an analyte (A) and an isotope labeled internal standard (IS) at known concentration (C)

$MF = \frac{peak\ area\ of\ blank\ matrix\ spiked\ after\ extraktion}{peak\ area\ of\ pure\ analyte\ solution}$ (Equation S3)

$IS\ normalized\ MF = \frac{MF\ of\ analyte}{MF\ of\ IS}$ (Equation S4)

$RE = \frac{peak\ area\ of\ blank\ matrix\ spiked\ before\ extraktion}{peak\ area\ of\ blank\ matrix\ spiked\ after\ extraktion}$ (Equation S5)

$IS\ normalized\ RE = \frac{RE\ of\ analyte}{RE\ of\ IS}$ (Equation S6)

Equations S3-6: Equations used to calculate the matrix factor (MF), the internal standard (IS) normalized MF, the recovery (RE) and IS normalized RE

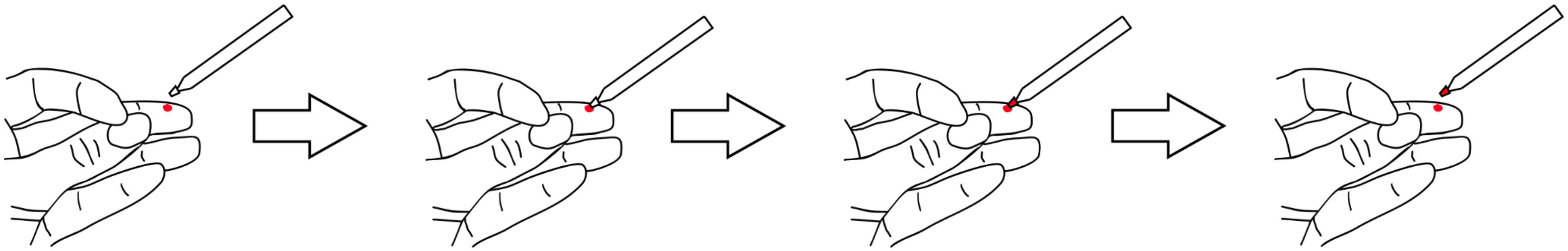

Figure S1: Sampling procedure for volumetric absorptive microsampling

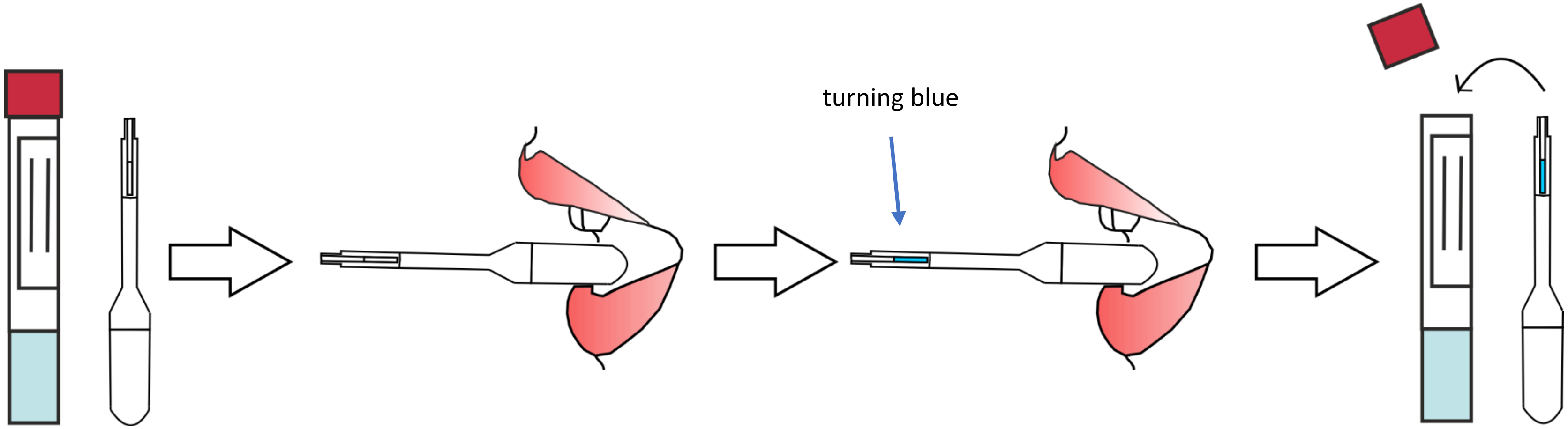

Figure S2: Sampling procedure for oral fluid

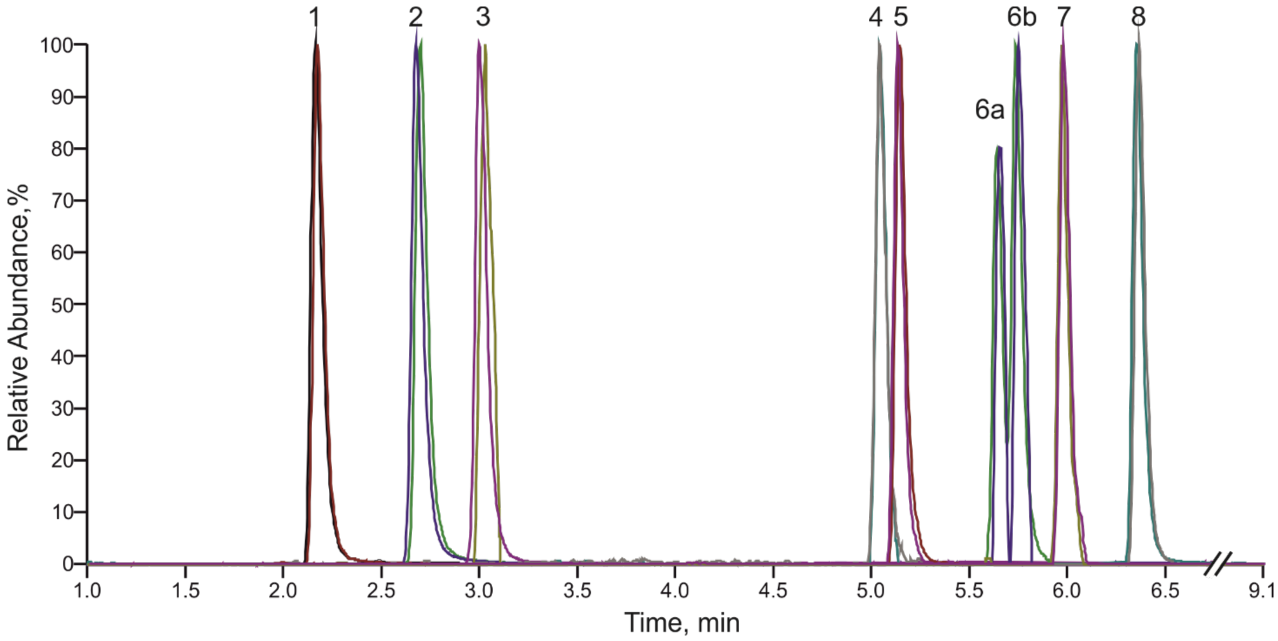

1	Ribociclib<br>*m/z* 435.2615	Ribociclib-d_6<br>*m/z* 441.2992
2	Abemaciclib<br>*m/z* 507.2791	Abemaciclib-d_8<br>*m/z* 515.3293
3	Palbociclib<br>*m/z* 448.2455	Palbociclib-d_8<br>*m/z* 456.2958
4	Letrozole<br>*m/z* 286.1087	Letrozole $^{13}C_2$, $^{15}N_2$<br>*m/z* 290.1095
5	Anastrozole<br>*m/z* 294.1713	Anastrozole $^{13}C_4$<br>*m/z* 298.1847
6a	Endoxifen (E)<br>*m/z* 374.2115	Endoxifen (E)-d_5<br>*m/z* 379.2428
6b	Endoxifen (Z)<br>*m/z* 374.2115	Endoxifen (Z)-d_5<br>*m/z* 379.2428
7	Exemestane<br>*m/z* 297.1849	Exemestane $^{13}C,d_3$<br>*m/z* 301.2071
8	Tamoxifen<br>*m/z* 372.2322	Tamoxifen-d_5<br>*m/z* 377.2636

Figure S3: Chromatographic separation of oral endocrine therapies and internal standards (quality control level 1) in plasma as sample matrix. All peaks are represented at 100% relative abundance.

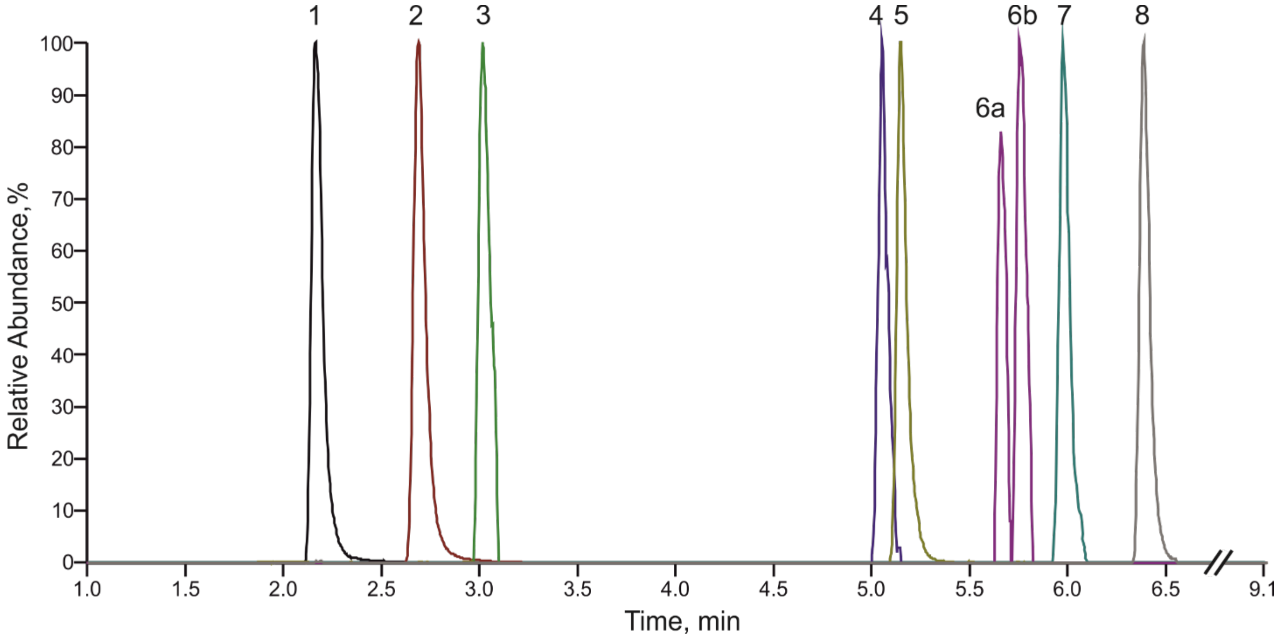

1	Ribociclib	*m/z* 435.2615
2	Abemaciclib	*m/z* 507.2791
3	Palbociclib	*m/z* 448.2455
4	Letrozole	*m/z* 286.1087
5	Anastrozole	*m/z* 294.1713
6a	Endoxifen (E)	*m/z* 374.2115
6b	Endoxifen (Z)	*m/z* 374.2115
7	Exemestane	*m/z* 297.1849
8	Tamoxifen	*m/z* 372.2322

Figure S4: Chromatographic separation of oral endocrine therapies at lower limit of quantification in plasma as sample matrix. For exemestane, the limit of detection is represented. All peaks are represented at 100% relative abundance.

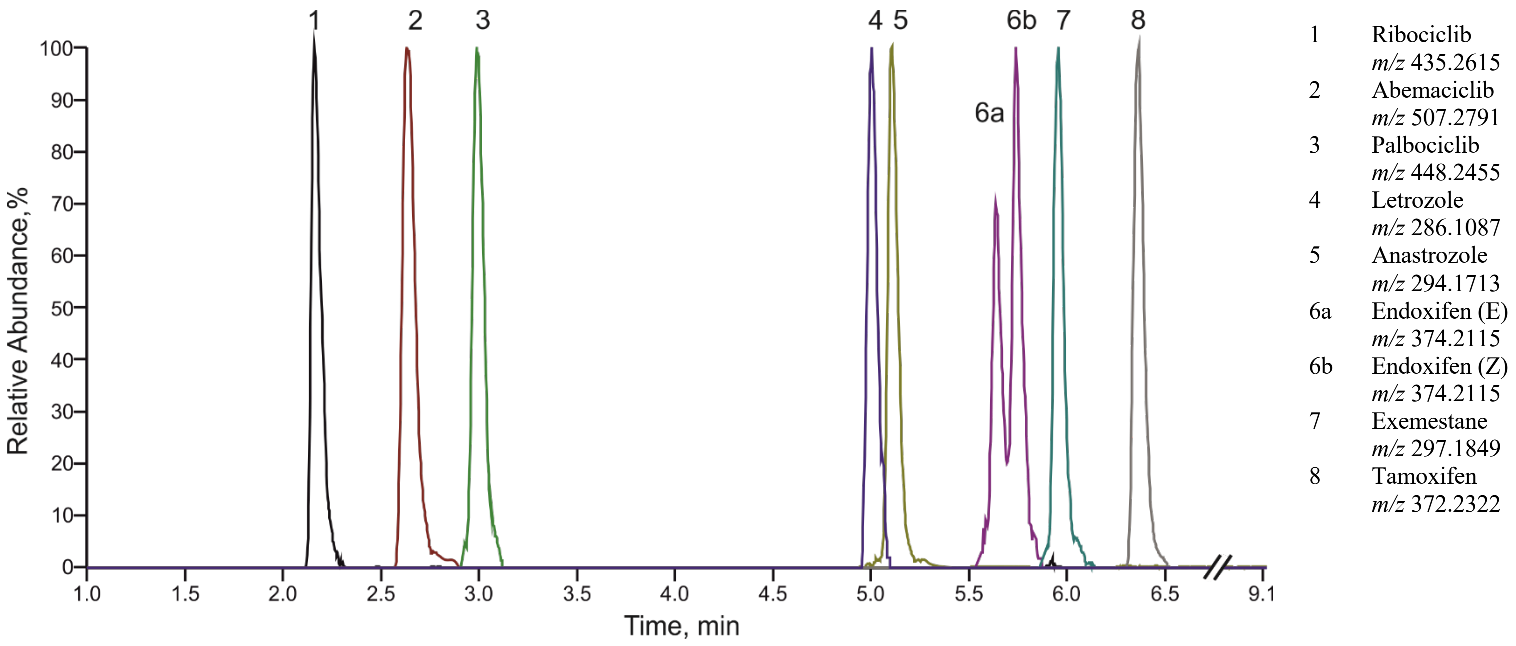

Figure S5: Chromatographic separation of oral endocrine therapies at lower limit of quantification in urine as sample matrix. For exemestane, the limit of detection is represented. All peaks are represented at 100% relative abundance.

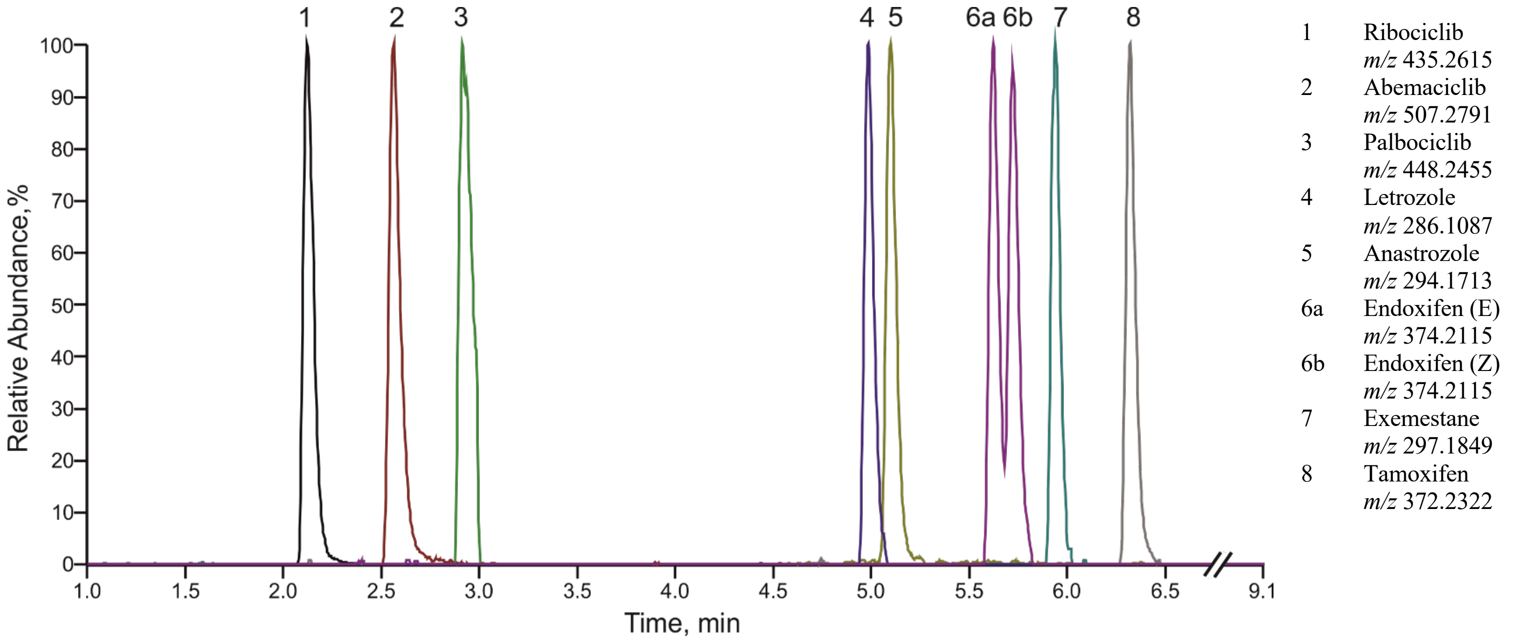

Figure S6: Chromatographic separation of oral endocrine therapies at lower limit of quantification in volumetric absorptive microsampling tips as sample matrix. All peaks are represented at 100% relative abundance.

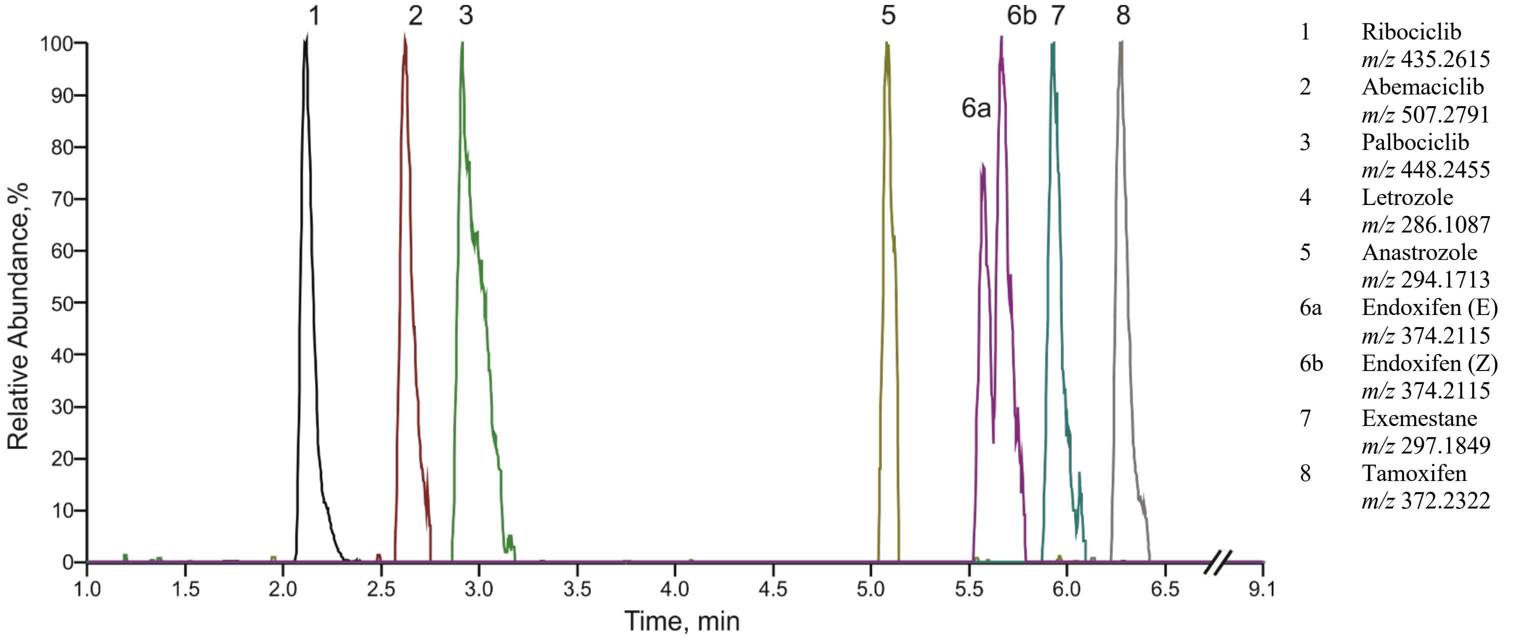

Figure S7: Chromatographic separation of oral endocrine therapies at lower limit of quantification in oral fluid (OF) as sample matrix. Letrozole is not detected. All peaks are represented at 100% relative abundance. Letrozole is not detected in OF.
